# Serial Spatial Transcriptomics Reveal Divergent Routes to Therapy Resistance in Metastatic Breast Cancer

**DOI:** 10.21203/rs.3.rs-9365657/v1

**Published:** 2026-04-15

**Authors:** Aaron Reid Doe, Aysegul Ors, Hugo Cros, Selim Sevim, Jinho Lee, Allison L. Creason, Gabriel Zangirolani, Joshua Rose, Isaac Youm, Furkan Ozmen, Tugba Y. Ozmen, Mark A. Dane, William Scott, Matthew E. K. Chang, Koei Chin, Laura M. Heiser, Jayne Stommel, Gordon B. Mills, Hisham Mohammed

**Affiliations:** 1. Cancer Early Detection Advanced Research Center, Knight Cancer Institute, Oregon Health & Science University, Portland, OR, USA; 2. Division of Oncological Sciences, Knight Cancer Institute, Oregon Health & Science University, Portland, OR, USA; 3. Department of Molecular and Medical Genetics, Oregon Health & Science University, Portland, Oregon 97201, USA; 4. Knight Diagnostic Laboratories, Oregon Health & Science University, Portland, OR, USA; 5. Department of Biomedical Engineering, Oregon Health & Science University, Portland, Oregon 97201, USA; 6. Brenden-Colson Center for Pancreatic Care, Knight Cancer Institute, OHSU

## Abstract

Metastatic solid tumors persist by evolving therapeutic resistance through complex, heterogeneous adaptive strategies that challenge standard precision medicine approaches. Current clinical decision making relies on bulk biomarkers, failing to resolve the spatial architecture and cellular contexts in which resistance mechanisms emerge. We present a patient-centric spatial framework, profiling 345,207 cells from four metastatic breast cancer patients across ten biopsies spanning personalized treatment courses of up to 3.5 years. By integrating probabilistic topic modeling with spatial deep learning, we observe fundamental principles of metastatic survival: pathway independence, microenvironment remodeling, and compensatory signaling. While these principles are universal, the underlying mechanisms are distinct: pathway independence manifested variously as the extinction of luminal identity, constitutive ESR1 activation, or spatial partitioning into drug-refractory invasive nests. Immune sanctuary was achieved through either genetic evasion mechanisms or physical exclusion via expanded fibroblast barriers. Compensatory transcriptional programs were engaged through rewired ligand-receptor networks and alternative survival pathway activation. These findings establish spatial profiling as a means to identify which mechanisms underlie each resistance principle in individual patients, enabling rational design of multi-axis combination therapies and earlier therapeutic decisions.

Despite major therapeutic advances, resistance to cancer treatment remains a significant challenge that prevents durable remissions in many patients, and in particular in metastatic disease^1^. Genetic clonal selection is a well-documented driver of tumor progression, as evidenced by therapy-induced emergence of subclones carrying new mutations that drive relapse^2,3^. Further, the frequent observation of clonal homogeneity in metastatic lesions^4,5^, despite continued phenotypic adaptation under therapy, highlights the limitations of a purely genetic model, pointing to nongenetic adaptations playing a role in disease progression and drug resistance^6^. In metastatic breast cancer, patients who progress through standard lines of endocrine and targeted therapy face increasingly empirical treatment decisions^7^, underscoring the critical need for methods to rapidly assess changing cell states within tumors and tailor therapy accordingly in near real-time.

Precision oncology trials have begun to address this challenge by coupling serial molecular profiling with adaptive treatment strategies^8–11^. The Serial Measurements of Molecular and Architectural Responses to Therapy (SMMART) program, for example, collects biopsies from metastatic lesions and performs integrative omics-based analyses to monitor tumor evolution and guide personalized therapy adjustments^12^. Initial results from a breast cancer patient with advanced disease from a SMMART-supported trial revealed remarkable heterogeneity across time, including discordant hormone receptor status and divergent genomic alterations, which corresponded with treatment adaptations^13^. Similarly, serial single-cell RNA sequencing of patients on aromatase inhibitor and CDK4/6 combination therapy has revealed actionable targets such as ERK/ERBB4 signaling associated with estrogen-independent resistance^11^. These studies underscore the value of longitudinal molecular profiling, but also highlight the need for spatially resolved, single-cell approaches to fully dissect intra-tumoral heterogeneity. Critically, the resistance mechanisms uncovered in such studies are often highly patient-specific, reflecting the unique mutational, transcriptional, and microenvironmental context of each tumor. This divergence argues that deep, per-patient molecular characterization rather than reliance on cohort-level recurrent features is essential when generalized therapeutic options have been exhausted.

While single-cell and spatial transcriptomics platforms offer the resolution needed to map distinct cell populations and states within their tissue context, current platforms are inherently noisy and sparse, with each region or cell capturing only a fraction of the transcriptome^14^. Furthermore, although single-cell transcriptomics and multi-omics have proven powerful for tracking and modeling developmental trajectories^15–19^, characterizing cancer cell states using sparse technologies is complicated by substantial inter- and intra-patient heterogeneity, and the absence of the consistent unidirectional transitions that are typical in developmental contexts.

To address these challenges, we employed a composite computational framework that deconvolves cells into continuous transcriptional programs via topic modeling (TITAN)^20^, identifies spatially cohesive tissue domains (STAGATE^21^), and anchors cell states in genetic ground truth through copy number alteration (CNA) inference from patient-matched whole-exome sequencing. This multi-pronged approach enables the consistent characterization of tumor evolution, stromal remodeling, and immune exclusion across patients and time, despite the noise and sparsity inherent in high-plex spatial imaging.

Here, we apply this framework to single-cell spatial transcriptomic profiling of serial metastatic biopsies from four breast cancer patients enrolled in SMMART-supported studies. Using the NanoString Technologies CosMx Spatial Molecular Imaging platform with the Human Universal Cell Characterization RNA Panel comprising 960 genes, we profiled 345,207 cells from four patients to quantify how tumor and microenvironmental states reorganize during therapy. We leverage deep longitudinal profiling to map patient-specific resistance trajectories, demonstrating that even tumors sharing a clinical subtype deploy fundamentally divergent escape strategies from interferon and antigen-presentation failure to the emergence of alternative transcriptional programs that replace lost dependencies.

## Results

### Longitudinal Spatial Transcriptomic Profiling of Metastatic Breast Cancer

**Figure 1.**
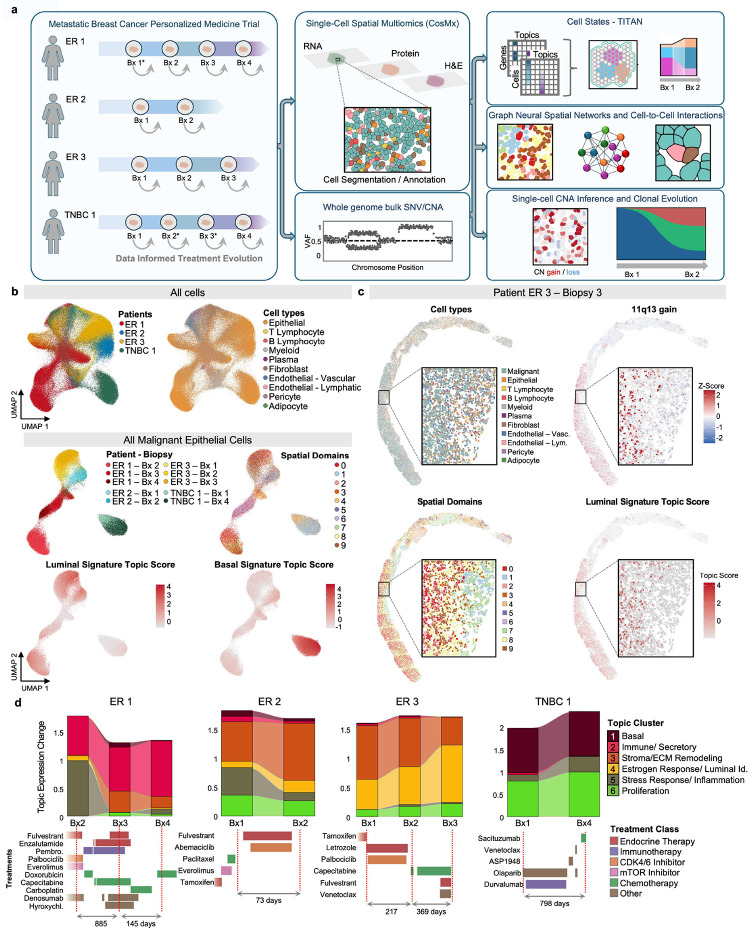
Overview of the study cohort and integrative workflow. **a.** Study overview showing four patients with estrogen receptor-positive (Patients ER 1, ER 2, ER 3) and triple-negative breast cancer (Patient TNBC 1) enrolled in the SMMART clinical trial with serial biopsies collected during adaptive therapy. The CosMx Spatial Molecular Imaging (SMI) platform was used for single-cell spatial transcriptomic and proteomic profiling, which were integrated with bulk whole-exome sequencing (WES). The computational workflow (right) utilizes TITAN for transcriptional state identification, alongside spatial domain mapping, ligand-receptor network analysis, and copy number inference to characterize tumor evolution across treatments and time. Asterisks (*) denote samples for which spatial transcriptomic data was not generated. **b.** UMAP visualizations. Top row: all cells from four patients (n=345,207) colored by patient of origin (left) and annotated cell type (right). Bottom row: subset of malignant epithelial cells (n=62,295) colored by patient and biopsy timepoint (top left), spatial domains identified by STAGATE (top right), and TITAN-identified topic scores for the Luminal (bottom left) and Basal (bottom right) signatures. **c.** Representative spatial visualization of patient ER 3, Biopsy 3 showing annotated cell types (top left), 11q13 copy number gain (top right), STAGATE-defined spatial domains (bottom left), and Luminal signature topic scores identified by TITAN (bottom right). **d.** Longitudinal changes in transcriptional topic cluster enrichment across malignant epithelial cells of sequential biopsies for each patient, illustrated as alluvial plots. Corresponding treatment regimens are annotated below each timeline and colored by treatment class. Biopsy timepoints are displayed at equal intervals for visualization clarity; actual elapsed time between timepoints is indicated numerically on each plot.

To elucidate the dynamics of tumor progression and therapeutic resistance, we performed longitudinal spatial transcriptomic profiling on four metastatic breast cancer patients enrolled in SMMART precision medicine studies. This cohort included three patients with estrogen receptor-positive (ER+) disease (patients ER 1, ER 2, and ER 3) and one with triple-negative breast cancer (patient TNBC 1), with serial biopsies collected during the course of therapy in each patient. To complement standard profiling in the SMMART platform, which included routine diagnostics, immunohistochemistry (IHC), multiplex IHC (mIHC), cyclic immunofluorescence (cycIF), bulk RNASeq, and whole-exome sequencing, we generated high-resolution spatial transcriptomic and protein data using the CosMx Spatial Molecular Imaging (SMI) platform. We profiled 10 tissue samples across the treatment timelines, resolving 345,207 single cells with a targeted 960-gene panel to capture the spatial architecture of the tumor microenvironment (Fig. 1a).

UMAP (Uniform Manifold Approximation and Projection) embedding of TITAN-derived topic scores^20^ across all cells from all patients and biopsies revealed distinct transcriptional states and cell lineages segregating into different regions of the latent space. (Fig. 1b, Extended Data E1a,c,e,f). Using established Human Breast Cancer Atlas (HBCA) markers^22–24^, we identified major cell lineages including T lymphocytes, B lymphocytes, myeloid cells, fibroblasts, endothelial cells (both vascular and lymphatic), pericytes, adipocytes, and epithelial populations (Fig. 1b). Importantly, TITAN-based clustering demonstrated reduced sensitivity to technical variation compared to standard approaches (Extended Data E1d-h). While Seurat-based UMAP embeddings showed strong influence of RNA count depth, with high-coverage cells clustering at projection extremes, TITAN embeddings exhibited more uniform cell distribution across clusters regardless of transcript coverage (Extended Data E1d,h). Quantitative assessment confirmed significantly lower coverage purity (see Methods) in TITAN clusters compared to Seurat (Extended Data E1g), indicating that technical depth was not a primary driver of clustering in the topic-based latent space.

Histopathological examination confirmed the presence of tumor tissue across all biopsies, with varying proportions of viable tumor, stroma, and adjacent normal tissue (Extended Data E2). To identify spatially coherent tissue domains, we applied STAGATE^21^, a graph attention autoencoder that integrates gene expression with spatial neighborhood information to learn domain-aware cell embeddings (Extended Data E3). STAGATE clusters cells based on both transcriptional similarity and physical proximity, enabling identification of tissue regions with shared microenvironmental context. While TITAN can optionally incorporate spatial coordinate information to identify regionally localized gene signatures (Extended Data E1b, i, j), for this study we relied on STAGATE-defined spatial domains to characterize the tissue architecture, using TITAN primarily for robust identification of transcriptional programs independent of spatial location.

Given the challenge of distinguishing malignant epithelial cells from normal cells using a targeted 960-gene panel, we leveraged matched exome sequencing data to identify tumor-specific chromosomal copy number alterations. Standard approaches such as InferCNV^25^ are limited when applied to sparse spatial transcriptomic data; instead, we tested whether large-scale chromosomal gains and losses identified from bulk sequencing of the tumor biopsy could be detected at single-cell resolution through targeted analysis of genes mapping to affected regions (see methods). This approach successfully identified distinct malignant populations in each patient based on characteristic copy number profiles (Extended Data E4). For example, an 11q13 amplification, a recurrent alteration in ER+ breast cancer^26^, was clearly detectable in individual cells from Patient ER 3 (Fig. 1c), enabling confident classification of malignant versus non-malignant epithelial populations.

We next grouped the 30 malignant cell topics (Supplementary Table 2) into six functional clusters based on pathway enrichment and gene composition (Extended Data E5): (1) Basal, (2) Immune/Secretory, (3) Stroma/ECM Remodeling, (4) Estrogen Response/Luminal Identity, (5) Stress Response/Inflammation, and (6) Proliferation (Extended Data E5). To examine how these programs changed with treatment, we quantified the relative abundance of each topic cluster across biopsies within each patient (Fig. 1d, Extended Data E5). ER 1, treated with endocrine therapy (Fulvestrant, Enzalutamide) along with immunotherapy (Pembrolizumab) and chemotherapy (Carboplatin, Capecitabine, Doxorubicin), showed a progressive shift away from Estrogen Response signatures (Cluster 4) toward increased Stress Response programs (Clusters 1 and 5) over 885 days of treatment. Patients ER 2 and ER 3, both ER+ and treated primarily with endocrine therapy, showed distinct trajectories: while they both maintained high Estrogen Response signatures despite fulvestrant treatment, ER 2 had increased Stromal and ECM remodeling while this signature decreased in patient ER 3 with a shift towards Proliferation signatures despite CDK4/6 inhibitor (palbociclib) treatment (Fig. 1d). Patient TNBC 1, who received chemotherapy, immunotherapy (durvalumab) and targeted agents (olaparib, ASP1948), exhibited dominant Basal and Proliferation signatures (Clusters 1 and 6) throughout, consistent with the aggressive biology of triple-negative disease.

These findings establish a framework for tracking spatially resolved transcriptional cell states in serial metastatic biopsies and reveal patient-specific patterns of tumor evolution under therapeutic pressure. The integration of topic modeling with single-cell spatial transcriptomics and CNA-based clone identification enables dissection of treatment-associated state transitions that may underlie resistance to targeted therapies.

### Acquired Mutations Drive Loss of Luminal Identity and Immune Evasion in Patient ER 1

**Figure 2.**
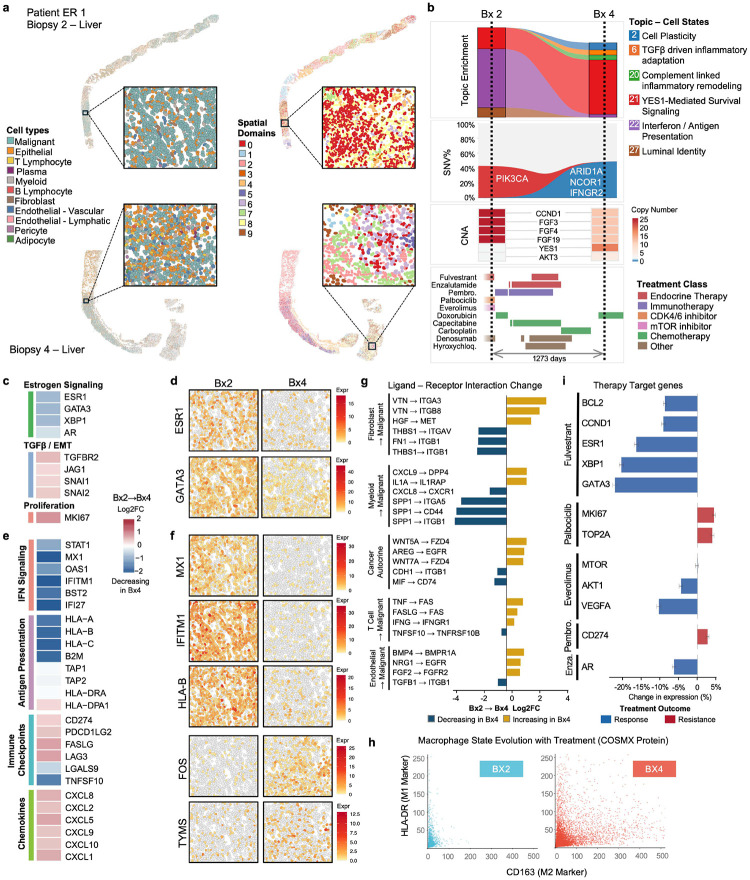
Longitudinal molecular evolution and immune microenvironment remodeling in Patient ER 1. **a.** Spatial characterization of longitudinal liver metastases (Bx2 and Bx4) from Patient ER 1. Panels display spatial projection of annotated cell lineages (left) and spatial domains identified by STAGATE (right), with high-resolution insets. **b.** Longitudinal molecular dynamics shown in vertical panels (from top to bottom): relative enrichment of selected transcriptional topics, including loss of Luminal Identity (Topic 27) and Interferon/Antigen Presentation (Topic 22) in Bx4; clonal evolution of driver mutations shown by Variant Allele Frequency (%VAF), highlighting PIK3CA in Bx2 and the acquired ARID1A, NCOR1, and IFNGR2 variants; a heatmap of selected Copy Number Alterations (CNAs); and the patient’s treatment timeline colored by therapeutic class spanning 1,273 days. **c.** Gene expression changes in Estrogen Signaling, TGFβ/EMT, and Proliferation between Bx2 and Bx4 shown as a Log2 fold change (Log2FC) heatmap. **d.** Spatial expression of ESR1 and GATA3 in Bx2 and Bx4, showing decreased expression in Bx4. **e.** Gene expression changes in IFN Signaling, Antigen Presentation, Immune Checkpoints, and Chemokines between Bx2 and Bx4 shown as a Log2FC heatmap. **f.** Spatial expression of immune pathway genes (MX1, IFITM1, HLA-B) showing decreased expression in Bx4, and functional markers (FOS, TYMS) showing increased expression in Bx4. **g.** Ligand-receptor interaction dynamics (Bx2→Bx4) depicted as a waterfall plot ranked by Log2FC. **h.** Macrophage phenotype marker shifts (M1/Anti-tumorigenic vs. M2/Pro-tumorigenic) depicted as a scatter plot. **i.** Treatment target gene expression changes between Bx2 and Bx4 shown as a bar plot; blue bars indicate response-associated and red bars indicate resistance-associated changes. For all Log2FC comparisons **(c,e,g)**, positive values indicate increased and negative values indicate decreased expression or interaction scores in Bx4 relative to Bx2.

To investigate mechanisms of treatment resistance in depth, we next focused on individual patient trajectories. Patient ER 1, diagnosed with ER+ invasive ductal carcinoma, was enrolled following progression on aromatase inhibitors^13^. Over the course of 1,273 days, the patient received multiple lines of therapy including endocrine agents (fulvestrant, enzalutamide), CDK4/6 inhibitors (palbociclib), mTOR inhibitors (everolimus), immunotherapy (pembrolizumab), and chemotherapy (doxorubicin, carboplatin, capecitabine) (Fig. 2a,b). We compared two liver biopsies collected 885 days apart (Bx2 and Bx4), excluding the non-liver biopsy (Bx3) to avoid site-specific confounding effects.

Spatial mapping of cell lineages revealed distinct tissue architecture in both biopsies, with cancer cells, fibroblasts, and immune populations organized into spatially coherent domains that corresponded to histological features on H&E staining (Fig. 2a, Extended Data E2). STAGATE analysis identified ten spatial domains in each biopsy, capturing regions of tumor nests, stromal compartments, and immune-enriched areas with distinct cellular compositions (Fig. 2a, Extended Data E3).

Whole Exome Sequencing (WES) results previously indicated^13^ that this patient harbored a recurrent 11q13 amplicon encompassing *CCND1, FGF3, FGF4, FGF19* (Fig. 2b, Supplementary Table 1, Extended Data E4c), a canonical alteration in ER+ breast cancer associated with endocrine resistance and poor prognosis^26,27^. The tumor exhibited significant spatiotemporal divergence; while the 11q13 amplification was ubiquitous, other clinically relevant alterations were confined to specific biopsies. Notably, Bx2 uniquely harbored a private PIK3CA E542K hotspot mutation (Supplementary Table 1), an oncogenic driver of the PI3K/AKT/mTOR pathway^28,29^, that was notably absent from all other biopsies (Supplementary Table 1). Subsequent biopsies (Bx3 and Bx4) demonstrated further clonal evolution, acquiring distinct amplicons on chromosome 18 encompassing *YES1* and *TYMS*. These alterations were accompanied by increased *TYMS* and *YES1* RNA expression following treatment with the TYMS inhibitor capecitabine, suggesting a selective pressure driving resistance^13,30,31^. Strikingly, Bx4 acquired multiple additional mutations absent in Bx2 including ARID1A (p.Q566* and p.S536F), NCOR1 (p.S2193*), and IFNGR2 (p.S39*) (Fig. 2b, Supplementary Table 1). *ARID1A* encodes a core subunit of the SWI/SNF chromatin remodeling complex; its loss in ER+ breast cancer disrupts FOXA1-mediated chromatin accessibility at luminal enhancers and drives resistance to endocrine therapy^32,33^. *NCOR1* functions as a nuclear corepressor that modulates ER transcriptional activity; its loss has been associated with tamoxifen resistance and poor prognosis^34,35^. The co-acquisition of these mutations suggests a convergent mechanism of ER pathway inactivation through epigenetic dysregulation.

Consistent with these genomic changes, transcriptional analysis revealed a marked shift in cancer cell states between biopsies. Topic 27 (Luminal Identity), characterized by high expression of *ESR1*, *GATA*3, and *CDH1* (Supplementary Table 2), was substantially reduced in Bx4 (Fig. 2b,c). Spatial mapping confirmed decreased *ESR1* and *GATA3* expression across tumor regions (Fig. 2d). Topics associated with cellular plasticity (Topic 2: *LEFTY1*, *S100A4*, *S100A2*), TGFβ-driven inflammatory adaptation (Topic 6: *TGFB1*, *AXL*, *FLT1*), and YES1-mediated survival signaling (Topic 21: *YES1*, *S100P*, *SQSTM1*) increased in abundance (Fig. 2b). This transcriptional rewiring was concurrent with high *MKI67* gene expression (Fig. 2c), indicating a transition from an estrogen-dependent luminal phenotype toward a plastic, proliferative state sustained by alternative survival pathways - a hallmark of endocrine therapy resistance^33^.

The acquired IFNGR2 nonsense mutation (p.S39*) was associated with a distinct immune evasion phenotype. *IFNGR2* encodes the signal-transducing subunit of the interferon-gamma receptor; its loss abrogates IFN-γ signaling, a critical pathway for tumor immunosurveillance and response to checkpoint inhibitors^36,37^. Concurrent with this mutation, we observed marked downregulation of STAT1, the principal shared effector of both Type I and Type II interferon signaling cascades. Because IFN-γ signaling positively regulates *STAT1* transcription, loss of *IFNGR2* is predicted to disrupt this feedback loop, depleting the signaling mediator required by both pathways. Consistent with this, the antigen-presentation and interferon-stimulated signatures (Topic 22 and Topic 25), enriched for interferon-stimulated genes (*STAT1*, *MX1*, *OAS1*, *IFITM1*, *IFITM3*) and MHC class I components (*HLA-A*, *HLA-B*, *HLA-C*, *B2M*, *TAP1*), were dramatically reduced in Bx4 (Fig. 2b,e, Supplementary Table 2). Notably, the genes most affected included both Type II-dependent and dual-pathway targets, suggesting that *STAT1* depletion effectively crippled interferon responsiveness beyond the Type II pathway alone. Spatial expression mapping confirmed loss of *MX1*, *IFITM1*, and *HLA-B* across the tumor (Fig. 2f), indicating a coordinated collapse of the antigen presentation machinery and interferon-stimulated gene programs that would render cancer cells invisible to cytotoxic T lymphocytes. The co-acquired *ARID1A* mutations may have further contributed to this immune signaling failure, as ARID1A loss has been shown to impair SWI/SNF-mediated chromatin accessibility at interferon-responsive regulatory elements, though the relative contribution of each mutation cannot be disentangled here.

Analysis of predicted ligand-receptor interactions revealed widespread changes in inferred cell-cell communication between biopsies (Fig. 2g). We observed a loss of interactions maintaining epithelial integrity in tumor cells, such as CDH1-ITGB1 (E-cadherin) and SPP1-mediated signaling axes (SPP1-CD44, SPP1-ITGA5) between myeloid and tumor cells. Conversely, the resistant tumor indicated at an acquired dependence on autocrine and paracrine growth-promoting pathways, prominently the HGF-MET and AREG-EGFR axes, as well as non-canonical Wnt signaling (WNT5A-FZD4, WNT7A-FZD4). These alterations suggest that as the tumor shed its luminal identity, it established alternative proliferative drives independent of ER signaling^38–41^.

Parallel to these tumor-intrinsic changes, the immune microenvironment underwent localized immunosuppressive remodeling. Macrophages exhibited a profound phenotypic shift from an HLA-DR^high^ anti-tumorigenic (M1-like) state in Bx2 to a CD163^high^ pro-tumorigenic (M2-like) state in Bx4 (Fig. 2h, Extended Data E6a). This polarization aligns with the loss of inflammatory signaling from the tumor (due to *IFNGR2* loss) and further reinforces the ‘cold’ immune niche^42^. Ripley’s L spatial analysis^43^ demonstrated reduced T cell aggregation in Bx4 compared to Bx2, suggesting dispersal of T cells away from organized immune structures. Protein-level validation using the CosMx Human Immuno-Oncology Protein Panel confirmed these findings (Extended Data E6b,c).

Assessment of therapeutic targets confirmed the complete decoupling of tumor growth from the initial driver pathways (Fig. 2i). While fulvestrant effectively engaged its targets as evidenced by the deep reduction of *ESR1*, *GATA3*, *XBP1*, and *BCL2*, this reduction did not translate to growth arrest. Instead, proliferation markers (*MKI67*, *TOP2A*) paradoxically increased despite concurrent CDK4/6 inhibition. Furthermore, resistance mediators displayed divergent survival signals: while upstream PI3K effectors like *AKT1* were suppressed, *MTOR* expression remained stable, and *CD274* (PD-L1) was upregulated, distinguishing the resistant state from the treatment-sensitive baseline.

Together, these findings demonstrate that ER 1’s tumor evolved through a synergy between specific genomic alterations and profound shifts in transcriptional state. This interplay resulted in a coordinate loss of luminal identity and a gain of cellular plasticity, facilitating evasion from immune surveillance and providing molecular mechanisms for the tumor to dynamically bypass successive lines of endocrine, cytotoxic, and immune-based treatments.

### Convergent ESR1 Mutations and Stromal Remodeling in Patient ER 2

**Figure 3.**
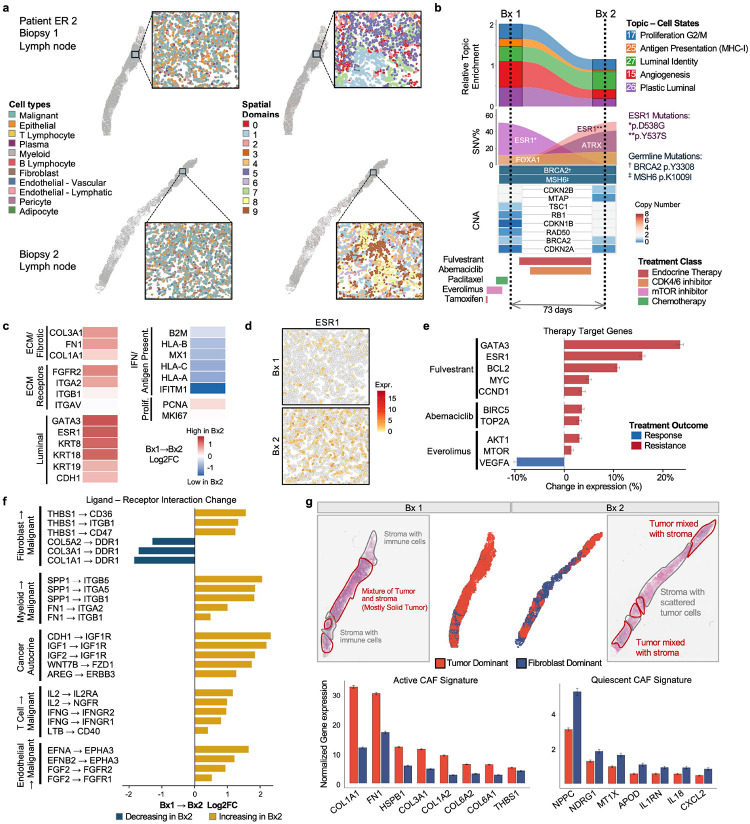
Convergent ESR1 mutations and stromal remodeling in Patient ER 2. **a.** Spatial projection of annotated cell lineages and spatial domains identified by STAGATE on longitudinal lymph node metastases (Bx1 and Bx2) from Patient ER 2, with high-resolution insets. **b.** Longitudinal molecular dynamics shown in vertical panels (from top to bottom): relative enrichment of selected transcriptional topics; clonal evolution of driver mutations shown by Variant Allele Frequency (%VAF), highlighting convergent ESR1 evolution with p.D538G in Bx1 replaced by p.Y537S in Bx2; germline *BRCA2* and *MSH6* mutations; a heatmap of selected Copy Number Alterations (CNAs); and the patient’s treatment timeline colored by therapeutic class over 73 days. **c.** Gene expression changes in ECM remodeling, Luminal identity, Antigen Presentation, and Proliferation between Bx1 and Bx2 shown as a Log2FC heatmap. **d.** Spatial expression of ESR1 in Bx1 and Bx2, showing increased expression in Bx2. **e.** Treatment target gene expression changes between Bx1 and Bx2 shown as a bar plot; blue bars indicate response-associated and red bars indicate resistance-associated changes. **f.** Ligand-receptor interaction dynamics (Bx1→Bx2) depicted as a waterfall plot ranked by Log2FC. **g.** Cancer-associated fibroblast (CAF) characterization. Top panels display H&E staining with pathological annotation and spatial domains categorized as “Tumor Dominant” or “Fibroblast Dominant.” Bottom bar plots quantify the differential expression of Active CAF signature genes (enriched in tumor dominant zones) versus Quiescent CAF signature genes (enriched in fibroblast dominant zones). For all Log2FC comparisons **(c,f)**, positive values indicate increased and negative values indicate decreased expression or interaction scores in Bx2 relative to Bx1.

Patient ER 2 is an ER+ breast cancer patient harboring germline BRCA2 p.Y3308* and MSH6 p.K1009I mutations which are associated with homologous repair^44,45^ and mismatch repair^46^ deficiencies respectively. Against this genomic background, the patient exhibited a distinctive pattern of tumor evolution characterized by convergent *ESR1* mutations and dramatic stromal remodeling. Following prior treatment with the selective estrogen modulator (SERM) tamoxifen, the mTOR inhibitor everolimus, and the taxane paclitaxel, a baseline lymph-node biopsy was obtained (Bx1). The patient was then treated with the selective estrogen receptor degrader (SERD) fulvestrant and the CDK4/6 inhibitor abemaciclib over a 73-day interval, after which a second lymph-node biopsy (Bx2) was collected for comparison (Fig. 3a,b, Extended Data E2, Supplementary Table 1).

Spatial analysis revealed dramatic changes in tissue architecture between biopsies. While Bx1 contained a mixture of solid tumor regions and stroma with immune cells, Bx2 showed extensive stromal expansion with tumor cells scattered within a fibroblast-rich matrix (Fig. 3a). STAGATE domain analysis captured this reorganization, identifying distinct regions of tumor mixed with stroma and stroma-rich areas with scattered tumor cells in Bx2 (Fig. 3a, Extended Data E3).

To investigate the molecular drivers of this remodeling, we tracked the genomic evolution of the tumor. Longitudinal sequencing revealed a striking example of convergent evolution via clonal replacement. While the tumor at the first metastatic site (Bx1) was dominated by an ESR1 D538G clone, the subsequent biopsy (Bx2) was comprised of a different clone harboring the ESR1 Y537S mutation (Fig. 3b, Supplementary Table 1). Both mutations are among the most prevalent ESR1 alterations in endocrine-resistant breast cancer and confer constitutive, ligand-independent receptor activation^47,48^. The detection of distinct mutations at the same functional domain in sequential biopsies suggests convergent evolution under therapeutic pressure, where independent subclones acquire different mutations to achieve the same functional outcome: escape from estrogen dependence. Despite the endocrine therapy (fulvestrant), *ESR1* expression increased in Bx2 (Fig. 3c,d). This upregulation is consistent with the enhanced stability and constitutive activity characteristic of the Y537S variant, and was accompanied by increased proliferative markers, confirming that the tumor had successfully bypassed the therapeutic blockade (Fig. 3c).

In contrast to Patient ER 1, undergoing dedifferentiation, ER 2 strengthened its luminal identity. Genes associated with the estrogen response and epithelial lineage (*ESR1*, *GATA3*, *KRT8*, *KRT18*, *CDH1*) and the Luminal Identity Topic (Topic 27) driven by canonical ER targets (*GATA3*, *ESR1*, *CCND1*, *CDH1*), increased in Bx2 (Fig. 3b–d). This lineage reinforcement coincided with a concurrent contraction of both Topic 26 (Plastic Luminal: *KRT19*, *PSAP, NEAT1*) and Topic 15 (Angiogenesis: *VEGFA*, *GDF15*). These patterns suggest the Y537S mutation effectively reinforced the tumor’s luminal state, potentially rendering it less dependent on plasticity or other proliferative pathways. Crucially, despite treatment with CDK4/6 inhibitors, this tumor remained proliferative, with Topic 17 (Proliferation: *MKI67*, *TOP2A*, *BIRC5*) persisting unchanged between biopsies (Fig. 3b), confirming the failure of therapy to induce cell cycle arrest.

A notable transcriptional change between the two lymph node biopsies was the expansion of extracellular matrix (ECM) components. Gene expression analysis revealed marked upregulation of fibrotic genes including *COL1A1*, *COL3A1*, *FN1*, and ECM receptors (*FGFR2*, *ITGA2*, *ITGB1*, *ITGAV*) in Bx2 (Fig. 3c). Topic analysis similarly indicated a reprogramming, identifying strong expansion of specific ECM remodeling signatures (Topic 19: *COL1A1*, *COL3A1*, *KRT19*, *FN1*, *IGFBP7*) (Supplementary Table 2, Extended Data E5a).

Assessment of therapeutic targets confirmed the functional dominance of this resistant state (Fig. 3e). While fulvestrant targets (*ESR1*, *GATA3*, *BCL2*) are typically suppressed in responsive tumors, for this patient, they were markedly upregulated, alongside oncogenic drivers like *MYC*. Crucially, this failure to suppress ER signaling translated to persistent cell cycle activity, with proliferation markers (*BIRC5*, *TOP2A*) remaining elevated despite concurrent CDK4/6 inhibition by abemaciclib. Additionally, we observed high expression of key components of the PI3K-mTOR survival pathway (*AKT1*, *MTOR*) in Bx2. This sustained activity is consistent with the absence of mTOR inhibition following discontinuation of everolimus (Fig. 3e).

Inference of ligand-receptor interactions highlighted a distinctly hyperactive signaling landscape likely fueled by uninhibited ER activity (Fig. 3f). Predicted cancer cell autocrine communication was dominated by the IGF pathway activation (CDH1→IGF1R, IGF1→IGF1R), potentially synergizing with constitutively active ESR1 mutations, alongside non-canonical WNT (WNT7B→FZD1) and ERBB (AREG→ERBB3) pathway engagement. Beyond autocrine stimulation, the tumor actively remodeled its immune niche. We observed a substantial increase in inferred myeloid-to-tumor signaling, with SPP1→ITGB5 (+2.2 Log2FC) representing the most upregulated signaling axis, indicating recruitment and activation of pro-tumorigenic SPP1+ tumor-associated macrophages^49,50^. In marked contrast to Patient ER 1, where immune evasion was driven by an acquired *IFNGR2* mutation, Patient ER 2 retained the capacity for immune sensing. T cell-to-tumor signaling increased across multiple axes, with IL2→IL2RA and both interferon receptor interactions (IFNG→IFNGR1, IFNG→IFNGR2) upregulated. This immune activation is consistent with the increased mutational burden and resulting neoantigenic load typically generated in BRCA2- and MSH6-deficient tumors with impaired DNA damage repair mechanisms^44–46,51^. However, despite this upstream immune activation, downstream interferon response genes (*MX1*, *HLA-B)* were concurrently downregulated (Fig. 3c), indicating a ‘disconnected’ signal where immune pressure was present but ineffective.

This functional decoupling was accompanied by extensive remodeling of the stromal compartment (Fig. 3f). Fibroblast-to-tumor communication showed a significant increase in thrombospondin signaling (THBS1→CD36, THBS1→ITGB1), suggesting that CAFs were actively signaling to the malignant cells to promote survival and adhesion. Interestingly, COL1A1→DDR1 interactions decreased, suggesting qualitative changes in the extracellular matrix (ECM) composition during this transition.

To investigate the functional state of the expanded fibroblast activity, we defined spatial domains according to tumor proximity and analyzed fibroblasts residing in tumor-dominant compared with fibroblast-dominant regions (Fig. 3g). Fibroblasts in the tumor dominant region exhibited higher expression of active cancer-associated fibroblast (CAF) markers including *COL1A1, FN1, HSPB1, COL3A1, COL1A2, COL6A2, COL6A1*, and *THBS1*, consistent with a myofibroblastic CAF (myCAF) phenotype often associated with ECM deposition and immune exclusion^52,53^. In contrast, those in the fibroblast dominant region retained a quiescent profile expressing markers such as *NPPC*, *NDRG1* and *CXCL2* suggesting spatial heterogeneity in fibroblast activation states (Fig. 3g). Neighborhood analysis confirmed that fibroblasts became increasingly prominent neighbors of tumor cells in Bx2, consistent with the formation of a desmoplastic barrier surrounding tumor nests (Fig. 3g, Extended Data E3).

These findings reveal a dual mechanism of treatment resistance in Patient ER 2: the convergent evolution of activating *ESR1* mutations, providing ligand-independent proliferation, and the expansion of an activated CAF population creating a dense fibrotic microenvironment. The functional decoupling observed between the T cell-to-cancer signal and downstream effector genes indicated that the Y537S-driven clone may facilitate immune evasion, potentially through the co-optation of a dense CAF-remodeled ECM that physically or chemically blunts effective immune pressure. The formation of CAF barriers has been implicated in immune exclusion and treatment resistance across cancer types^52^, and their emergence in ER 2 suggests that stromal remodeling may represent an underappreciated mechanism of adaptive resistance in breast cancer that protects metabolically active, drug-resistant tumor nests from both pharmacological blockade and immune surveillance.

### GATA3 Gain-of-Function and Intratumor Heterogeneity in Patient ER 3

**Figure 4.**
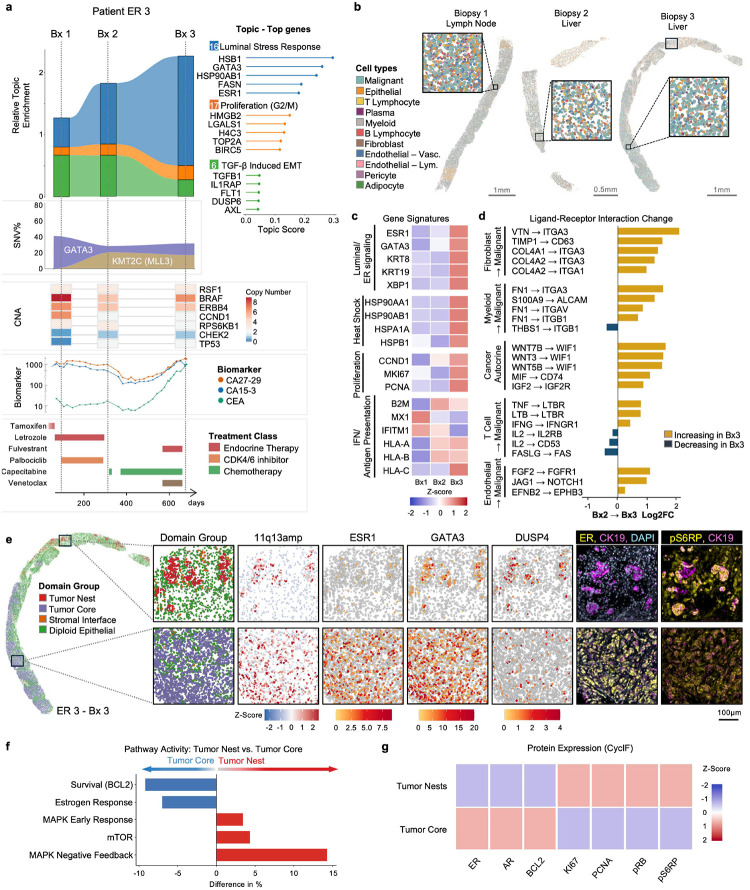
GATA3 gain-of-function and intratumor heterogeneity in Patient ER 3. **a.** Longitudinal molecular dynamics across three biopsies in Patient ER 3 (Bx1: Lymph Node; Bx2 and Bx3: Liver) shown in vertical panels (from top to bottom): relative enrichment of selected transcriptional topics; clonal evolution of GATA3 (C321fs) and KMT2C SNVs shown by Variant Allele Frequency (%VAF); a heatmap of selected Copy Number Alterations (CNAs) including BRAF amplification; longitudinal tracking of clinical biomarker levels (CA27–29, CA15–3, CEA); and the patient’s treatment timeline colored by therapeutic class over >600 days. **b.** Spatial projection of annotated cell lineages for Bx1, Bx2, and Bx3, with high-resolution insets. **c.** Gene expression across Luminal/ER signaling, Heat Shock, Proliferation, and Interferon/Antigen Presentation shown as a Z-score normalized heatmap across biopsies. **d.** Ligand-receptor interaction dynamics (Bx2→Bx3) depicted as a waterfall plot ranked by Log2FC; positive values indicate interactions gained and negative values indicate interactions lost in Bx3 relative to Bx2. **e.** Spatial characterization of tumor architecture in Biopsy 3 (Bx3). Spatial domains were categorized into functional zones: Tumor Nests, Tumor Core, Stromal Interface, and Diploid Epithelial. Comparative analysis of selected Tumor Nest versus Tumor Core regions highlights distinct features, including 11q13 copy number amplification, mRNA expression of *ESR1*, *GATA3* and *DUSP4* (MAPK negative feedback regulator), and cyclic immunofluorescence (cycIF) staining for estrogen receptor (ER), phosphorylated ribosomal protein S6 (pS6RP, mTOR effector), cytokeratin 19 (CK19, luminal epithelial) and DAPI (nuclei). **f.** Transcriptional pathway activity comparison between Tumor Nest and Tumor Core regions depicted as a bar plot showing the percentage difference in activity scores; the Tumor Core (blue) is enriched in survival and estrogen response pathways while Tumor Nests (red) are enriched in MAPK and mTOR pathways. **g.** Differential protein expression (cycIF) between Tumor Nest and Tumor Core regions shown as a Z-score heatmap.

ER 3, an ER+ breast cancer patient, harbored a GATA3 C321fs frameshift mutation, a recurrent alteration in luminal breast cancers that results in gain-of-function activity through enhanced protein stability and altered transcriptional output^54,55^ (Supplementary Table 1). Over 600 days of treatment including letrozole, palbociclib, tamoxifen, fulvestrant, capecitabine, and venetoclax, we collected three sequential biopsies: one from lymph node (Bx1) and two from liver metastases (Bx2 and Bx3) (Fig. 4a,b, Extended Data E2). Serum biomarker levels (CA15–3, CEA, CA27–29) tracked disease progression, with biopsies collected at key inflection points in the clinical course (Fig. 4a).

Longitudinal topic analysis revealed substantial transcriptional changes across biopsies. The final liver biopsy (Bx3) showed marked enrichment in the Luminal Stress Response topic (Topic 16) (Fig. 4a, Supplementary Table 2), characterized by elevated expression of luminal markers (*ESR1, GATA3, KRT8, KRT19, XBP1*) and heat shock proteins (*HSP90AB1, HSP90AA1, HSPA1A, HSPB1*), along with an increased Proliferation Topic (Topic 17) and increased proliferation gene expression (*CCND1, MKI67, PCNA*) (Fig. 4c). Concurrently, interferon and antigen presentation signatures (*HLA-A, HLA-B, HLA-C, B2M, MX1, IFITM1*) increased over time. The co-occurrence of proteotoxic stress markers (XBP1, HSPs) and upregulated antigen presentation genes is consistent with the reported ability of cellular stress responses to enhance MHC-I antigen presentation^56^.

Analysis of ligand-receptor interactions revealed a rewired microenvironment characterized by enhanced stromal support and immune evasion (Fig. 4d). We observed substantial increases in several signaling axes, most notably integrin-mediated ECM interactions (VTN→ITGA3, COL6A1→ITGA3, FN1→ITGA3) in fibroblasts and myeloid lineages. This physical scaffolding was accompanied by soluble pro-survival signaling with fibroblasts providing TIMP1→CD63 axis support, while the tumor simultaneously reinforced its own survival through autocrine MIF→CD74 signaling. Crucially, the immune interface displayed an uncoupling of inflammatory signaling and cytotoxicity: while inflammatory cytokine interactions (IFNG→IFNGR1, TNF→LTBR) increased in Bx3, the apoptotic interaction FASLG→FAS was sharply downregulated. These findings suggest that while the tumor remained exposed to pro-inflammatory cytokines, it established a multi-layered defense against T-cell mediated apoptosis by downregulating death receptor engagement and reinforcing stromal-mediated survival signals.

Beyond inter-biopsy differences, the final liver biopsy (Bx3) revealed striking intratumor heterogeneity that illustrated the spatial architecture of an advancing tumor front. STAGATE-based domain analysis distinguished four distinct spatial domains: a central Tumor Core, peripheral Tumor Nests, a Stromal Interface, and surrounding Diploid Epithelial liver tissue (Fig. 4e). The Tumor Core, comprising the bulk of the tumor mass, exhibited high expression of *ESR1* and *GATA3*, consistent with the gain-of-function *GATA3* mutation driving luminal differentiation under endocrine therapy^54,55,57,58^. In contrast, small Tumor Nests invading the surrounding diploid liver parenchyma displayed a markedly different transcriptional profile with: absent *ESR1* expression, elevated MAPK negative feedback (*DUSP4, DUSP6*), increased mTOR pathway activity, and higher proliferation (Fig. 4e–g). Notably, these invasive nests were supported by a stromal interface domain enriched for activated fibroblasts.

To investigate the spatial heterogeneity driving tumor evolution, we focused on several micrometastatic tumor nests within a single patient biopsy, as these proliferative fronts likely represent the forefront of tumor progression. We confirmed these regions were genomically altered tumor tissue by detecting a recurrent 11q13 amplification (encompassing *CCND1* and *RPS6KB1*) across the sampled areas (Fig. 4e). Crucially, spatial analysis revealed that while both the Tumor Core and Nests maintained high expression of *GATA3* (Fig. 4e), domain–specific differences emerged at the signaling and transcriptional levels. Compared to the Tumor Core, the Tumor Nests exhibited a marked pathway switch characterized by reduced estrogen response and survival signaling, alongside enhanced activation of MAPK and mTOR survival pathways (Fig. 4f). This shift may be driven in part by the GATA3 C321fs gain-of-function mutation, which has been shown to reprogram transcriptional targets including MAPK pathway components operating independently of ESR1^54,55,59^. By maintaining high *GATA3* levels, these nests retained their luminal identity (*KRT8*, *KRT19*) despite the loss of *ESR1* expression, suggesting a state of receptor-independent luminal differentiation supported by the mutant *GATA3* background. Of note, bulk genomic analysis of the biopsy identified a BRAF p.L18V mutation alongside a focal copy-number gain on chromosome 7 in Bx2 supplemented with a SND1-BRAF fusion in Bx3, providing a broader genomic context for activation of the MAPK pathway within the tumor^60–62^.

Cyclic immunofluorescence (cycIF) imaging validated these transcriptional findings at the protein level. The ER-positive Tumor Core showed strong ER and CK19 staining, while invasive Tumor Nests lacked ER expression but demonstrated elevated phospho-S6 ribosomal protein (pS6RP), confirming mTOR pathway activation (Fig. 4e,g). Quantitative protein analysis confirmed the distinct molecular profiles of these spatial domains: the Tumor Core was characterized by elevated ER, AR and BCL2 expression, consistent with a luminal, BCL-2 dependent survival program, while Tumor Nests showed higher Ki67, PCNA, pRB, and pS6RP, reflecting enhanced proliferative and mTOR activity (Fig. 4g).

These findings in patient ER 3 illustrate how spatial transcriptomics can resolve intratumor heterogeneity that would be masked in bulk analyses. The coexistence of an ER-positive Tumor Core with ER-negative, GATA3-driven, invasive Tumor Nests characterized by MAPK/mTOR pathway activation within a single biopsy has profound therapeutic implications: endocrine therapy combined with BCL-2 inhibition would target the core but spare the invasive front, while MAPK or mTOR inhibitors might address the invasive population but leave the luminal core untreated. This spatial heterogeneity underscores the need for combination strategies that address the full spectrum of molecular vulnerabilities present within a single tumor.

### Transcriptional Stability in Triple-Negative Breast Cancer Despite Therapeutic Pressure

**Figure 5.**
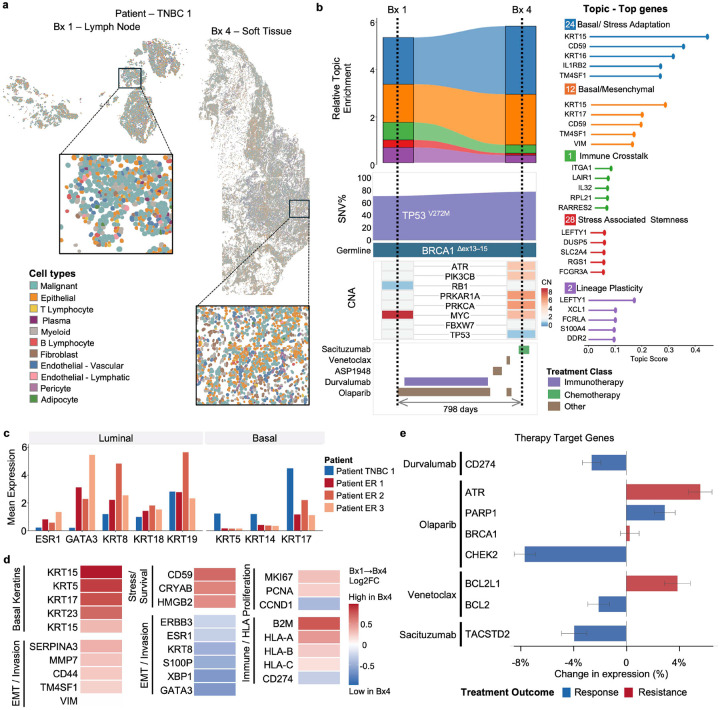
Transcriptional stability in triple-negative breast cancer despite therapeutic pressure (Patient TNBC 1). **a.** Spatial projection of annotated cell lineages from patient TNBC 1 on longitudinal biopsies from lymph node (Bx1) and soft tissue (Bx4), with high-resolution insets. **b.** Longitudinal molecular dynamics between Bx1 and Bx4 shown in vertical panels (from top to bottom): relative enrichment of selected transcriptional topics; clonal evolution of the TP53 driver mutation (V272M, %VAF); a heatmap of selected Copy Number Alterations (CNAs); and the patient’s treatment timeline colored by therapeutic class over 798 days. **c.** Comparative mean expression of canonical Luminal and Basal markers across the cohort, highlighting the distinct basal phenotype of patient TNBC 1 relative to the ER+ patients (ER 1, ER 2, ER 3). **d.** Gene expression changes in Basal Keratins, EMT/Invasion, Stress/Survival, and Immune signaling between Bx1 and Bx4 shown as a Log2FC heatmap; positive values indicate increased and negative values indicate decreased expression in Bx4 relative to Bx1. **e.** Treatment target gene expression changes between Bx1 and Bx4 shown as a bar plot; blue bars indicate response-associated and red bars indicate resistance-associated changes.

Patient TNBC 1, a triple-negative breast cancer (TNBC) case in our cohort, provided a biological contrast to the ER+ patients. This patient’s genetic background was characterized by a somatic TP53 V272M mutation (70% VAF) and a high-confidence germline deletion of BRCA1 exons 13–15^63^ (Fig. 5b, Supplementary Table 1). Over 798 days, the patient received intensive multimodal therapy including immunotherapy (durvalumab), PARP inhibition (olaparib), an investigational agent targeting NRP1 (ASP1948), BCL-2 inhibition (venetoclax), and antibody-drug conjugate therapy (sacituzumab govitecan) (Fig. 5b). We compared a lymph node biopsy (Bx1) with a soft tissue biopsy (Bx4) collected after these multiple lines of systemic treatment.

Spatial mapping revealed similar tissue architecture across both biopsies, with cancer cells organized in dense clusters surrounded by stromal and immune populations (Fig. 5a, Extended Data E2). In contrast to the ER+ patients, patient TNBC 1’s tumor exhibited a distinctly basal transcriptional profile, with high expression of basal keratins (*KRT5, KRT14, KRT17*) and absence of luminal markers (*ESR1, GATA3, KRT8, KRT18, KRT19*) (Fig. 5c), characteristic of TNBCs^64^.

Remarkably, the transcriptional landscape remained largely invariant between the two available biopsies, despite extensive therapeutic pressure. Topic analysis revealed consistent enrichment for basal/stress adaptation programs (Topics 24, 12), immune crosstalk (Topic 1), and stress-associated stemness signatures (Topic 28), with only subtle shifts in relative abundance (Fig. 5b, Supplementary Table 2, Extended Data E5). The genomic profile was similarly conserved: the germline BRCA1 deletion and somatic TP53 mutation persisted at high VAF and CNA including MYC amplification and RB1 loss remained stable across biopsies (Fig. 5b, Supplementary Table 1).

Gene-level analysis revealed modest changes in expression consistent with treatment-induced stress responses. Basal keratins (*KRT15, KRT5, KRT17, KRT23*) and Epithelial-mesenchymal transition (EMT)/invasion markers (*SERPINA3, MMP7, CD44, TM4SF1, VIM*) showed slight increases, while stress/survival genes (*CD59, CRYAB, HMGB2*) were modestly upregulated (Fig. 5d). Proliferation markers (*MKI67, PCNA, CCND1*) and immune/HLA genes (*B2M, HLA-A, HLA-B, HLA-C, CD274*) exhibited variable but limited changes. Analysis of drug target expression revealed that while the patient was showing molecular hallmarks of response to immunotherapy (durvalumab) and antibody-drug conjugate (ADC) therapy (sacituzumab), the tumor was developing resistance to the targeted inhibitors through different mechanisms. Specifically, it had bypassed olaparib by leveraging a focal gain in ATR, a critical compensatory pathway for maintaining replication fork stability in BRCA1-deficient contexts^65^, while simultaneously bypassing venetoclax by shifting its survival dependency to BCL-XL^66^.

Patient TNBC 1’s tumor maintained a broadly stable basal phenotype between the two available biopsies, in stark contrast to the extensive transcriptional reprogramming observed in the ER+ cases. This transcriptional stability, despite long-term multimodal treatment, suggests that resistance was largely associated with pre-existing programs rather than adaptive acquisition, consistent with the aggressive and treatment-refractory biology described in basal-like subtypes^67^.

## Discussion

Unlike primary tumors, therapy-resistant metastatic breast cancers are characterized by accelerated evolutionary trajectories driven by sequential pharmacological pressures^68^. The convergence of progressive genomic instability and profound cellular plasticity enables these tumors to continuously bypass selective bottlenecks, resulting in highly heterogeneous and unique mechanisms of survival. In this study, we attempt to understand the spatiotemporal dynamics of this process. We profiled 345,207 cells across ten serial biopsies from four patients treated over up to 3.5 years and find that pathway independence and alternative compensatory signaling, as well as immune/microenvironment remodeling are key themes. While these are well established hallmarks of cancer^69^, we find that the mechanisms are profoundly patient and context-specific. Mapping the evolutionary trajectories of treatment-refractory tumors is essential to uncover acquired vulnerabilities and ultimately define shared therapeutic strategies that transcend patientlevel heterogeneity.

In our three ER+ patients, we observe non-redundant modes of escape from ER dependence. In Patient ER 1, mutations targeting key ER cofactors, particularly ARID1A led to a coordinated collapse of luminal identity, accompanied by rewiring toward plasticity and stress-adaptation programs, consistent with the known ability of endocrine pressure to select for epigenetic and transcriptional states that no longer require canonical ER circuitry. Patient ER 2 exemplifies the ligand-independent progression characteristic of late-stage ER+ disease. Notably, we observe a distinct clonal replacement where one activating ESR1 ligand-binding domain (LBD) mutation is substituted for another (D538G by Y537S). This evolutionary shift preserves, and even amplifies, luminal transcriptional programs, enabling the tumor to sustain proliferation despite concurrent CDK4/6 blockade. This convergent selective pressure on ESR1 aligns with the established role of ligand binding domain mutations as primary drivers of endocrine resistance and their marked enrichment in metastatic lesions^47,48^. While emerging next-generation selective estrogen receptor degraders (SERDs) and PROTACs have been developed to specifically target these mutant conformations and partially restore therapeutic efficacy^70,71^, the spatial dynamics of this clonal succession highlight the adaptability of the disease. Patient ER 3 is driven by a GATA3 C321fs mutation, an alteration established to reprogram and hyper-activate luminal transcriptional networks^57,58^. While the tumor core maintains this strengthened luminal, ER-high profile, our spatial approach resolves a divergent, highly aggressive phenotype at the advancing front. We map multiple micrometastatic nests infiltrating adjacent normal liver tissue; strikingly, these invasive compartments retain their robust GATA3 and luminal identity but exhibit complete ER loss, pivoting their dependency to altered MAPK/mTOR signaling. Understanding these properties, unique to the emerging tumor front, can potentially enable therapeutic interventions at an earlier stage.

From an immune evasion perspective, Patient ER 1 exemplifies active, genetically driven remodeling of the tumor microenvironment. The acquisition of a truncating *IFNGR2* mutation coincides with a localized, precipitous collapse of both interferon-stimulated and antigen-presentation transcriptional programs. Because *IFNGR2* encodes the essential signal-transducing subunit of the IFN-γ receptor complex, this loss-of-function mutation abrogates downstream JAK/STAT signaling, effectively blinding the mutant clone to immune sensing. Crucially, this intrinsic signaling collapse drives a profound spatial reorganization characterized by reduced T cell aggregation and a striking shift toward immunosuppressive macrophage polarization. Consistent with CRISPR screens demonstrating that *IFNGR2* mutations are highly selected for and confer a survival advantage specifically within mixed-clonal environments^36^, our data captures how this mutation actively engineers an immune-privileged niche *in situ*. Conversely, Patient ER 2 maintains intact receptor-ligand immune communication but circumvents immune clearance through profound stromal remodeling. By establishing a fibroblast-rich, ECM-dense microenvironment, the tumor creates a physical barrier that restricts immune effector access despite ongoing immune pressure. This spatial dichotomy dictates distinct therapeutic strategies. The stromal exclusion phenotype suggests that anti-tumor immunity could be successfully unlocked by combining standard immunomodulation with therapies targeting CAF programs and extracellular physical barriers^52,53,72^.

While large, cross-sectional atlases are invaluable for defining generalizable disease paradigms^73^, our study is intentionally designed as a deep longitudinal case series to resolve the mechanistic granularities required for precision oncology. Although our cohort size inherently limits population-level generalizations, its definitive strength lies in profound spatiotemporal depth. By repeatedly sampling across critical clinical inflection points, we decode the precise, within-patient evolutionary logic that single-timepoint or bulk-sequencing studies inevitably obscure^68^. Much like TCGA-era bulk profiling struggled to capture low-frequency, context-specific vulnerabilities, our spatial mapping reveals that critical resistance programs are fundamentally geographically sequestered. Because these actionable targets are sustained by highly localized microenvironments, they remain effectively invisible to standard bulk assays, even when present at high local abundance.

Several technical constraints must frame our interpretation. The reliance on a targeted CosMx panel inherently restricts pathway inference. Additionally, serial sampling across disparate anatomic sites dictates that apparent clonal dynamics reflect a combination of true evolution and spatial sampling bias, where distinct metastatic niches may exert unique microenvironmental forces that drive site-specific adaptation. Nonetheless, while our findings are primarily associative and warrant future functional validation, such as modeling *IFNGR2* loss or CAF-driven exclusion in patient-matched systems, our computational framework successfully overcomes the noise and sparsity of high-plex spatial data. By integrating topic modeling with spatial domain learning and grounding malignant states in matched genomic alterations, we transform complex spatial coordinates into coherent, actionable resistance narratives at single-patient resolution.

Ultimately, therapy-resistant tumors survive by solving key universal constraints: shedding original dependencies, building immune sanctuaries, and activating compensatory proliferation. Consequently, personalized therapeutics must evolve beyond empirical drug layering by leveraging technologies that allow us to target the disease earlier and with higher precision.

## Methods

### Patient Cohort, Sample Collection and Clinical Assays

This study was approved by the Oregon Health & Science University (OHSU) Institutional Review Board (IRB). Biospecimens and data for patients ER 1, ER 2, ER 3, and TNBC 1 B4 were acquired and analyzed under the OHSU IRB-approved protocols Molecular Mechanisms of Tumor Evolution and Resistance to Therapy (MMTERT, IRB#16113) and Reconstructing the Tumor Genome in Peripheral Blood (IRB#10163). Biospecimen and data for patient TNBC 1 Bx 1 were acquired and analyzed under a pilot study combining the PARPi olaparib and the PD-L1 antibody durvalumab (IRB#1829)^63^. Participant eligibility was determined by the enrolling physician and informed written consent was obtained from all subjects. Biopsy sites were selected by physicians based on imaging, prioritizing accessibility, and patient safety, with a preference for the largest lesion to maximize tumor yield. Biopsy specimens were immersed in formalin within 3 minutes of collection to stabilize protein expression and retain post-translational modifications. Samples underwent formalin fixation for approximately 12 hours per ASCO/CAP guidelines prior to paraffin embedding (FFPE). Histopathological features were annotated by board-certified pathologists on H&E stained serial sections. Whole exome sequencing (WES), GeneTrails solid tumor panel sequencing, cyclic immunofluorescence, and serum biomarker measurements (CA15–3, CEA, CA27–29) were performed as part of the SMMART analysis platform. Sample collection, processing, and analytical protocols for these assays are described in detail in Johnson et al.^13^ and Labrie et al.^63^

### CosMx Spatial Molecular Imaging: RNA

CosMx spatial molecular imaging (SMI) data was generated from formalin-fixed paraffin-embedded (FFPE) breast cancer tissue sections from 4 patients: ER 1, ER 2, ER 3, and TNBC 1. Each patient contributed biopsies at different treatment timepoints (ER 1: Bx2, Bx3 and Bx4; ER 2: Bx1 and Bx2; ER 3: Bx1, Bx2 and Bx3, TNBC 1: Bx1 and Bx4). 5 μm FFPE tissue sections were embedded in the slides (Superfrost plus, ThermoFisher) under 37°C nuclease-free water conditions and dried at 37°C for 2 hours prior to baking at 60°C overnight. Tissue samples were then deparaffinized twice in xylene for 5 min, then twice in 100% Ethanol for 2 min, then dried at 60°C for 5 min. All jars and trays used after this step were cleaned with RNase Away to prevent RNA degradation. Tissue samples were briefly adjusted in diethyl pyrocarbonate treated water (DEPC water, Thermo Fisher Scientific) at 100°C and antigen retrieval was immediately performed at 100°C in a steamer for 15 min. After antigen retrieval, tissue samples were rinsed once with DEPC water, washed once in 100% ethanol for 3 min and dried at room temperature (RT) for 1 hour. The adhesive incubation frame was applied around the tissue section and samples were incubated with Proteinase K at 3 μg/ml at 40 °C for 30 min in the hybridization chamber oven (HyBEZ II, ACDBio). After rinsing twice with DEPC water, samples were incubated in 1:400 diluted fiducials in 2x saline sodium citrate and Tween-20 (2x SSC-T) buffer for 5 min at room temperature, then washed once with 1xPBS for 5 min. After fiducial incubation, samples were fixed with 10% neutral-buffered formalin (NBF) for 1 min, then washed twice with NBF stop buffer (0.1 M of Tris-base and glycine) for 5 min per wash and 1x PBS for 5 min. Fixed tissue was stabilized with 100 mM N-succinimidyl (acetylthio) acetate (NHS-acetate, ThermoFisher) in NHS-acetate buffer for 15 min at RT then washed once in 2× SSC for 5 min. The CosMx^™^ Human Universal Cell Characterization RNA Panel (comprising 960-plex RNA ISH probes; NanoString Technologies) was applied and samples were hybridized at 37°C for 16–18 hours. After hybridization, slides were washed twice with 50% formamide in 2x SSC at 37 °C for 25 min to remove non-specific probes, then washed twice with 2x SSC for 2 min each at RT. Samples were incubated with morphological and cell segmentation markers (PanCK/CD45 and CD298/B2M) for 1 hour, washed three times in 1x PBS for 5 min each and incubated with DAPI nuclear stain for 15 minutes at RT. After two washes with 2x SSC for 5 min each, the incubation frame was removed and a flowcell was applied to each slide prior to loading onto the CosMx instrument. Sample images were scanned and 0.5 mm × 0.5 mm FOVs were placed in a continuous grid that covered the entire tissue area. After 16 cycles of fluorescent reporter hybridization and imaging to read out the RNA barcode, raw data were processed using the AtoMx Spatial Informatics Platform for cell segmentation and transcript detection. This pipeline yielded single-cell spatial coordinates and transcript counts for each tissue section, which were subsequently compiled into a Seurat v5 object comprising 345,207 cells across 960 gene targets in all tissues assayed. Cells were annotated by label transfer from a recent breast cancer atlas defining epithelial subtypes^24^. Cell-type annotation was further refined by distinguishing copy number alteration (CNA) containing epithelial cells from diploid epithelial populations.

### CosMx Spatial Molecular Imaging: Protein

Adjacent 5 μm sections of the same FFPE samples were used to generate matched spatial protein expression data. Tissue samples were deparaffinized with xylene twice for 5 min and sequentially rehydrated with 100% ethanol (twice for 10 min), 95% ethanol (twice for 5 min), 70% ethanol for 5min. Antigen retrieval (AR, Nanostring Technologies) buffer in the Coplin jar was preheated in the pressure cooker. Sample slides were briefly rinsed once with 1x PBS and immediately transferred to the preheated AR buffer. Samples were incubated at high pressure for 15 min, then cooled for 25 min at room temperature (RT). After 3 washes with 1x PBS for 5 min each, tissue samples were incubated with Buffer W (Patient ER 1) for 1 hour at RT. The primary antibody panel (CosMx^™^ Human Immuno-Oncology Protein Panel, 67 targets) was prepared by mixing the protein probe pool with morphological (PanCK/CD45) and cell membrane (CD298/B2M) markers. Samples were incubated with the antibody mix at 4°C for 16–18 hours and then washed with TBS-T 3 times for 10 min each. Fluorescent fiducials (Nanostring Technologies) were prepared via sequential vortexing and sonication, and applied to samples for 5 min at RT. Following a 5 min wash in 1x PBS for 5 min, samples were post-fixed with 10% NBF for 15 min at RT. Samples were washed three times with 1x PBS for 5min each and incubated with 100mM NHS-acetate buffer at RT for 15 min in the dark. After one wash with 1x PBS for 5 min, samples were stained with DAPI for 10min at RT and a flowcell was then applied to each slide for loading onto the CosMx instrument. Imaging was performed on the CosMx instrument using the same 0.5 mm × 0.5 mm FOV grid defined for the matched RNA sections. After 16 cycles of fluorescent reporter hybridization and imaging, raw data were processed using the AtoMx Spatial Informatics Platform for cell segmentation and protein quantification, yielding single-cell spatial protein expression profiles for 67 targets across 48,898 cells in Bx2 and 45,605 cells in Bx4 for Patient ER 1. These cells averaged over 17,000 protein counts per cell.

### TITAN: Single-Cell Topic Modeling

Spatial transcriptomics data generated from the CosMx^™^ Human Universal Cell Characterization RNA Panel were analyzed using TITAN, a topic modeling framework based on Latent Dirichlet Allocation (LDA)^20^. In this framework, individual cells are treated as discrete documents and genes as words, with transcript counts representing word frequencies. The algorithm produces two output matrices: a cell-topic distribution matrix, which serves as a low-dimensional embedding for interrogating transcriptional patterns and cellular relationships, and a gene-topic distribution matrix, used to define the biological context of each topic. Cells were filtered for a minimum of 200 transcript counts, resulting in a high-confidence dataset of 140,763 observations with an average of 320 transcript counts across 182 unique genes. From this baseline, a subset of 62,341 malignant cells was identified as epithelial cells (annotated by label transfer from HBCA^24^) harboring copy number alterations (CNAs) inferred with our *spatialCNA* package (see methods). Input features consisted of the 960-gene CosMx panel, and the count matrix was normalized using centered log-ratio (CLR) transformation. Models were trained across a range of 10 to 100 topics in increments of 10, using default hyperparameters (*α* = 50, *β* = 0.1, iterations = 500, burnin = 250). The optimal number of topics for each run was determined by evaluating the inflection point of the rate of perplexity change across models. A topic number of 30 was determined for the malignant cell topics used in this study.

### Performance Benchmarking and Sparsity Analysis

To evaluate TITAN’s robustness against technical dropouts and sparsity, performance was benchmarked against standard Seurat pipelines using the unfiltered dataset of 345,207 cells. A topic number of 60 was determined as previously described. The TITAN cell-topic matrix and the Seurat principal component analysis (PCA) matrix were embedded into Uniform Manifold Approximation and Projection (UMAP) space and colored by sequencing coverage to visually assess coverage-driven clustering. Louvain clustering was independently applied to both matrices, and coverage distributions across the resulting clusters were visualized via boxplots. To calculate coverage purity, cells were stratified into five equal coverage bins, and the purity of these bins within the latent spaces was computed. Higher coverage purity is interpreted as the dimensionality reduction being driven by sequencing depth rather than biological signal.

### Topic Model Interpretation and Clustering

Cell-topic distributions were visualized as heatmaps and the biological identity of each topic was defined by the top genes ranked by topic score, interpreted against known signaling pathways and the literature. To facilitate interpretation across all malignant cells from all patients and biopsies, topic values were averaged within each patient-timepoint stratum and represented as a condensed heatmap (Extended Data E5a). To identify overarching biological programs, topics were grouped into context-specific clusters. Briefly, a k-nearest neighbor (kNN) graph (*k*=5) was constructed from the gene-topic matrix, followed by Louvain clustering (resolution = 0.9). Intra-patient, inter-biopsy dynamics were summarized by averaging topic cluster scores per patient-biopsy group and visualizing the results as alluvial plots. Topic-specific alluvial plots were generated by applying the same averaging procedure to selected individual topics.

### Spatial Data Integration in TITAN

Tissue spatial context was incorporated into the topic model by encoding each cell’s position as a proximity-weighted feature vector (Extended Data E1b). Spatial centroids were first defined by applying k-means clustering (*k*=200) to the two-dimensional cellular coordinates (*x,y*). The spatial embedding for each cell was then computed as a proximity score to each centroid by subtracting each centroid distance from the maximum distance present in the dataset, such that cells receive the highest scores for their nearest centroids. For each cell, scores were thresholded to retain only the top *n* closest centroids (representing the top 5% by default). The retained values were subsequently normalized by a scaling factor (default = 10) to calibrate the weight of the spatial context. This procedure generated a 200-dimensional spatial feature vector per cell, which was concatenated to the gene-cell count matrix prior to executing TITAN’s *runLDA* function. The resulting topics were categorized into three classes: gene-restricted topics (analogous to standard TITAN topics), spatial-restricted topics (excluded from downstream analysis due to absence of biological interpretability) and spatially-integrated gene topics (“spatial topics”). These spatial topics capture associations between tissue location and gene expression, interpretable as spatially localized transcriptional networks.

### Spatial Domain Identification using STAGATE

Spatially coherent tissue domains were identified using STAGATE^21^, a graph attention autoencoder that integrates gene expression with spatial neighborhood information to learn domain-aware cell embeddings. All analyses were performed using default parameters as described in the original publication.

### Copy Number Alteration Inference

Copy number alterations (CNA) were inferred directly from CosMx spatial transcriptomics expression data using *spatialCNA*, an R package we developed for CNA detection in sparse targeted panels. Conventional expression-based CNA tools (InferCNV, CopyKat, SCEVAN) require hundreds of genes per chromosome arm and fail on targeted spatial platforms profiling ~1,000 genes. *spatialCNA* circumvents this by defining targeted probe regions from patient-specific clinical genetics reports, mapping CosMx panel genes to each region based on genomic coordinate overlap (GRCh38/hg38). For each cell, probe scores were computed as z-score normalized mean expression of genes within each probe region, referenced against non-cancer cells (fibroblasts, endothelial, and immune cells) as a diploid baseline. Scores for deletion probes were inverted so that positive values consistently indicate CNA-concordant expression. Probes were considered validated if they achieved a Bonferroni-corrected Wilcoxon p < 0.05, effect size > 0.3, and direction consistent with the expected event type. A per-cell CNA burden score was computed by summing validated probe z-scores, and cells were classified as CNA-altered versus diploid using Gaussian Mixture Models (*mclust*, variable variance). *spatialCNA* was applied to all four patients across serial biopsy timepoints. Gain events were consistently more detectable than losses, with validated gain probes achieving effect sizes of 0.31–1.35 and strong class separation across all patients (Cohen’s d > 2.5). Recurrent validated events included 11q13 gain (*CCND1*; Patients ER 1, ER 2, ER 3), 8q gain (*MYC*; Patients ER 2, ER 3, TNBC 1), 1q gain (Patients ER 1, ER 2), and 17q gain (Patients ER 2, TNBC 1).

### Ligand-Receptor Pair Database and Interaction Scoring

Ligand-receptor (LR) pairs were obtained from OmniPath, a comprehensive meta-database that integrates curated interactions from multiple resources including CellPhoneDB, CellChat, and NATMI. The OmniPath LR interaction set was filtered to pairs where both the ligand and receptor genes were present in the CosMx 960-gene panel, yielding 462 testable LR pairs. LR interactions were evaluated across biologically motivated cell-type pairing contexts: Fibroblast-to-Malignant, Myeloid-to-Malignant, Malignant-to-Myeloid, TCell-to-Malignant, Malignant-to-TCell, Endothelial-to-Malignant, Cancer Autocrine, and Malignant-to-Endothelial. For each LR pair within a given context, an interaction score was computed as the product of the mean log-normalized ligand expression in sender cells and the mean log-normalized receptor expression in receiver cells, per patient and timepoint (minimum 10 cells per group). When a context involved multiple lineages (e.g., vascular and lymphatic endothelial), expression values were combined using a weighted mean proportional to cell counts. The Fold change (FC) between two biopsies was calculated as Log2(Score_Late / Score_Early) with a pseudocount of 0.001.

## Supplementary Material

Supplementary Files

This is a list of supplementary files associated with this preprint. Click to download.

- Table1AllPatientsGeneticData.xlsx
- Table2allCosmxcancerTITANtopGenes.xlsx
- ExtendedDataFigureswLegends.pdf

Extended Data Figures E1-E6

Supplementary Tables

- Table_1_All_Patients_Genetic_Data.xlsx

Genomic alterations across all patients and biopsies.

Sheet 1 (CNA): Copy number alterations per patient and biopsy, including gene, alteration type, copy number range, chromosomal location, and source platform. Sheet 2 (SNV): Single nucleotide variants per patient and biopsy, including gene, nucleotide change, protein change, variant allele frequency, and source platform. CNA, copy number alteration; SNV, single nucleotide variant; VAF, variant allele frequency; VUS, variant of uncertain significance. Tier classifications follow AMP/ASCO/CAP somatic variant guidelines.

- Table_2_allCosmx_cancerTITAN_topGenes.xlsx

Top 50 genes ranked by topic score for each TITAN-identified transcriptional topic in malignant cells.

Each column represents one of 30 TITAN-identified topics (Topic 1–30) derived from malignant cells across all patients and biopsies. Rows list the top 50 genes in descending order by gene-topic score.

## Data Availability

The CosMx SMI raw data used in this study is deposited in the Gene Expression Omnibus (GEO) database (https://www.ncbi.nlm.nih.gov/geo/) under accession code GSE325216 (reviewer access token: qfapiooaxzihhwj).

## Code Availability

The code used to perform the analyses and generate the figures in this study is available in the GitHub repository at: https://github.com/HishamMohammedLab/Serial_Spatial_Transcriptomics_BC_paper.

The *spatialCNA* package is an open-source tool provided under the MIT license and can be accessed at: https://github.com/HishamMohammedLab/spatialCNA.
